# Hallux Limitus: Exploring the Variability in Lower Limb Symmetry and Its Connection to Gait Parameters—A Case–Control Study

**DOI:** 10.3390/bioengineering12030298

**Published:** 2025-03-14

**Authors:** Natalia Tovaruela Carrión, Ricardo Becerro-de-Bengoa-Vallejo, Marta Elena Losa-Iglesias, Daniel López-López, Juan Gómez-Salgado, Javier Bayod-López

**Affiliations:** 1 Faculty of Nursing, Physiotherapy and Podiatry, University of Seville, 41009 Seville, Spain; ntovaruela@us.es; 2 Faculty of Nursing, Physiotherapy and Podiatry, Complutense University of Madrid, 28040 Madrid, Spain; ribebeva@ucm.es; 3 Faculty of Health Sciences, Universidad Rey Juan Carlos, 28922 Alcorcón, Spain; marta.losa@urjc.es; 4 Research, Health and Podiatry Group, Department of Health Sciences, Faculty of Nursing and Podiatry, Industrial Campus of Ferrol, Universidade da Coruña, 15403 Ferrol, Spain; 5 Department of Sociology, Social Work and Public Health, Faculty of Labour Sciences, Universidad de Huelva, 21004 Huelva, Spain; salgado@uhu.es; 6 Safety and Health Postgraduate Program, Universidad Espíritu Santo, Guayaquil 092301, Ecuador; 7 Applied Mechanics and Bioengineering Group (AMB), Aragon Institute of Engineering Research (I3A), Centro de Investigación Biomecánica en Red CIBER-BBN, Universidad de Zaragoza, 50009 Zaragoza, Spain; jbayod@unizar.es

**Keywords:** foot, hallux limitus, instability, gait analysis, gait patterns

## Abstract

Hallux limitus pathology is defined as a limitation of the dorsiflexion movement of the first toe without degenerative involvement of the first metatarsophalangeal joint, which produces pain and generates functional impairment, especially in the propulsive phase of gait. It is very common to find this pathology in adulthood accompanied by other compensations at a biomechanical level as a consequence of blockage of the main pivot in the sagittal plane. The aim was to determine the symmetry index that occurs in dynamics affiliated with other gait parameters in subjects with and without hallux limitus. A total of 70 subjects were part of the sample, and these were separated into two groups, each consisting of 35 subjects, depending on whether they had bilateral hallux limitus or if they were healthy subjects. In this study, a platform was used to assess the load symmetry index and walking phases. The results showed significant differences in the symmetry index for lateral load (*p* = 0.023), the initial contact phase (*p* = 0.003), and the flatfoot phase (*p* < 0.001). The adults who had bilateral hallux limitus exhibited changes in the symmetry index during the lateral load as well as in the initial contact and flatfoot contact phases, demonstrating increased instability when compared to individuals with normal feet.

## 1. Introduction

Hallux limitus (HL) is explained as a painful pathology for the subject who suffers from it, in which a functional limitation of the first metatarsophalangeal joint (IMTFJ) in dorsiflexion (DF) movement can be detected during the take-off phase without degenerative alteration of the IMTFJ [1]. For normal propulsion to exist, in healthy subjects, an amplitude of 58° of DF is required by the hallux [2,3]. HL is a common foot condition suffered by adults, and it is distinguished by a rise in impact with age [4]. The etiology and treatment of HL are poorly understood; it is considered a pathology of multifactorial causes [3,5]). One of the explanations for HL is found in the windlass mechanism [5], first described by Hicks [6] (HICKS, 1954); this mechanism allows the medial arch to be raised and compacted through the plantar fascia, preparing it for the take-off phase, at which time the foot must be in a locked position in order to transmit all the force and allow elevation or take-off. Taking this into account, any modification in the plantar fascia that alters the windlass mechanism can produce HL [5,6,7,8].

First ray hypermobility, longer metatarsals, a raised first metatarsal, an inward-pointing big toe, being female, and having a family history have all been mentioned as risk factors [3,9]. Among the most common signs presented by subjects suffering from HL are hyperkeratotic skin lesions over the plantar/plantar–medial area of the hallux (pinch callus) as well as hyperkeratosis under the second metatarsal head as a consequence of load transfer that may or may not be associated with metatarsalgia [10].

The IMTFJ has an important role in the entire biomechanics of gait, fundamentally in the propulsive phase, which is why the presence of HL produces alterations in gait patterns because of the blockage of the third rocker [4,5,6,7,8,9,10,11]. The third rocker begins when the heel starts to lift off the ground and ends when the entire foot is lifted off the toe area to begin the swing phase of the foot. At this point, good mobility is required on the part of the metatarsophalangeal joints so that overload does not occur in this zone [12,13]. According to Dananberg and his theory of sagittal plane facilitation [14], this dynamic limitation of the IMTFJ during gait can lead to compensation in other joints, which alters the normal gait pattern and generates greater energy expenditure due to faster, inefficient movement. This compensation can extend beyond the foot, affecting the knees, hip, and even the lumbar region [10,12,15,16]. This secondary compensation, produced with the objective of improving joint movement in the propulsive phase of walking, causes a variation in plantar pressures that can be analyzed with a baropodometric platform, which also serves to analyze the balance and sway of the body [2,13,17].

Foot instability and metabolic disorders could have a considerable impact on the quality of life of the subjects affected, and it is greater in women than men [18,19,20]. Baropodometry is one of the most useful tools for analyzing posture, balance, and stability [21]. This system provides information about the state of our feet and how it influences the health of the lower limbs [13,22]. For all the characteristics described previously, the population with HL has a higher chance of falling due to issues with the movement in their walking pattern, along with joint looseness during the final and middle stages of walking [23]. Furthermore, metabolic alteration, such as increased body weight, can increase plantar loading, which represents a greater risk of foot injuries. This increased load during continuous walking in people with excess body weight has been related to a higher incidence of pathological gait patterns, decreased mobility, and, in the future, greater likelihood of disability [24]. Given all of this, our hypothesis was that subjects with HL would be less stable because of uneven posture, which would create changes in the center of pressure, increasing the contact area compared to adults with normal feet. The aim of this investigation was to examine the dynamic symmetry index in the inferior limbs by comparing individuals with and without HL.

## 2. Materials and Methods

### 2.1. Design and Sample

The sample consisted of 70 subjects (11 men and 59 women) recruited in a biomechanical laboratory in Coruña, aged between 21 and 39 years (mean age 25.8 years) who were divided into two groups: the case group, consisting of 35 overweight subjects with bilateral HL, and the control group, consisting of another 35 subjects with healthy feet. To calculate the sample size, G*Power Version: 3.1.9.3 software (Heinrich-Heine-Universität Düsseldorf, Düsseldorf, Germany) was used with a large effect size of 0.80, an α error probability of 0.05, with a β level of 20% and a two-tailed hypothesis, resulting in a sample size of 70 participants calculated (35 subjects in each group).

The inclusion criteria for the case group were as follows: being between 18 and 64 years of age, not having a general health history, not having previous lower limb surgeries, having bilateral HL, signing the informed consent form to participate in this study, and having the ability to understand the instructions for participating in this study. For the control group, the inclusion criteria were the same, except that, in this case, the subjects had to have healthy feet with a bilateral neutral foot. The exclusion criteria for both groups were as follows: being under 18 years of age or over 64 years of age, being pregnant or breastfeeding, having any alteration or pain in the foot, having lower limb surgery, not having HL, refusing to sign the informed consent, or not having the capacity to understand the instructions for participating in this study.

This study was conducted in accordance with the ethical principles set out in the Declaration of Helsinki and in Law 14/2007 (July 4) on Biomedical Research. Data collection complied with the principles of confidentiality and anonymity, following the Organic Law 3/2018 of 5 December on the protection of personal data and guarantee of digital rights. This research was carried out following all the Strengthening the Reporting of Observational Studies in Epidemiology (STROBE) guidelines [25]. This study has been approved by the Research Ethics Committee at the University of A Coruña, under record number 2024-033.

### 2.2. Methods

This study was carried out by a podiatrist with experience in biomechanics, who first conducted an interview with the participants to collect data. Each subject was then weighed and measured to assess the body mass index (BMI) (Macdonald, 1986). The hallux limitus test was performed through the clinical test described by Dananberg [14], which has been reviewed by authors to determine its reliability [10,26]. For this, the patient was placed in a supine position with the ankle in a neutral position, and the clinician had to apply a passive force to the plantar area of the head of the lower IMTH, avoiding plantar flexion and with the other active hand performing DF of the hallux until reaching the maximum amount of movement and the examiner feeling the plantar flexion of the first metatarsal, and to measure it, a two-branch goniometer was used. The test is considered negative when the clinician manages to perform dorsal flexion and positive when it is not possible or greater force is needed to perform DF of the IMTFJ.

**Figure 1:**
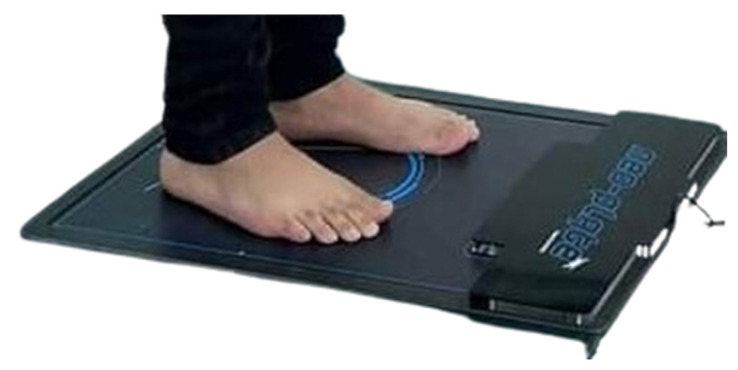
Neo-Plate portable pressure mapping platform.

In this research, a baropodometric platform was used, a validated device for foot diagnosis [27], following the same protocol as Becerro de Bengoa Vallejo et al. [28]. The Neo-Plate pressure platform is equipped with resistive sensors and has a useful surface area of 40 × 40 cm, allowing the patient to fully assess their gait (Figure 1). Its flat design, with a thickness of only 8 mm, facilitates its integration into various clinical environments and its transport. The acquisition frequency is adjustable between 100 and 500 Hz. By measuring the pressure in different areas of the foot (forefoot, midfoot, and hindfoot) during each step, the platform can calculate the symmetry index of the gait, comparing the relationship between the limbs. If the symmetry index shows values close to 100%, it means that the patient is walking with a balanced distribution of forces between both feet. On the contrary, a lower index would indicate significant asymmetries, which may be associated with muscle weakness, mobility disorders, or neurological disorders (Neo-Plate, Herbitas, Spain).

The data were analyzed with Neo-Plate^®^ Version 1.12.28.0 for Windows software. Autocalibration was performed before each use, and subjects were asked to walk a few minutes prior to starting the data analysis to adjust their walking speed. The analysis was considered valid when a heel-strike–toe-off sequence was executed and the walking speed was consistent [29,30]. Three trials were recorded for each foot to obtain a more reliable result, and the average was calculated using the software for analysis. If any analysis was very dispersed, it was classified as outlier, and a new analysis of the subject was performed [2].

In this study, the symmetry index developed by Robinson et al. [31] has been applied to evaluate the impact of certain pathological conditions, such as HL, on foot function, using the equation $SI=\frac{Xr-Xi}{0.5|Xr|+|Xi|}$ · 100, where SI is the symmetry index, Xr is the variable recorded for the right leg, and Xi the variable recorded for the left leg. This technique is the most used and cited in studies on gait symmetry [32,33].

For body weight, dynamic pressure maps were produced, which provided anterior, posterior, medial, and lateral load, initial contact phase (ICP), flatfoot contact phase (FFCP), and forefoot phase (FFP). All the foot variables recorded were compared and interpreted using percentages and meters per second.

### 2.3. Statistical Analysis

To perform the statistical analyses, a statistical package for Windows, IBM SPSS Statistics 29.0.01.0 (Armonk, NY, USA), was used. The Shapiro–Wilk test was used for analyzing the normality of the dynamic symmetry index variables (*p* > 0.05). The Mann–Whitney “U” test was used for non-parametric phenomena to contrast groups with and without HL. In the case of the parametric data, these are described as the mean ± standard deviation and ranges of minimum to maximum values. Regression modeling for determining if BMI independently influences gait patterns was performed, and partial eta squared was used to measure the effect size of different variables in ANOVA models. The value for partial eta squared ranges from 0 to 1, where values closer to 1 indicate a higher proportion of variance that can be explained by a given variable in the model after accounting for variance explained by other variables in the model. The following rules of thumb are used to interpret values for partial eta squared: 0.01: small effect size; =0.06: medium effect size; and 0.14 or higher: large effect size. NeoPlate software Version 1.12.28.0 for Windows was used to acquire the dynamic symmetry index produced for each foot.

## 3. Results

None of the variables showed normal distribution (*p* < 0.05).

### 3.1. Sociodemographic Data

Of the 70 total participants, the control group could only be made up of 35 subjects, who were those who presented HL, and the other 35 were part of the control group. The participants were between 21 and 39 years old. A high proportion of the subjects were overweight, with a total weight of 72.26 ± 14.28 kg and a total body mass index (BMI) of 26.62 ± 5.48 kg/m^2^, with significant differences between the groups being observed in both (Table 1).

### 3.2. Main Outcome Measure Data

The results of the measurements for the total sample, which have also been differentiated by groups (with HL and healthy controls), are shown in Table 2, where it can be observed that several variables analyzed showed significant differences. This table also includes the effect size, where it can be observed that for the variables medial and lateral load for both the right and left lower limb, the effect size was close to 0.8. Also noteworthy is the value obtained for the symmetry index in the FFP, where, in addition to obtaining a statistically significant result (*p* < 0.001), the effect size is 0.791.

## 4. Discussion

One of the relevant parameters for the evaluation of gait in patients with HL is the symmetry index. This index refers to the relationship between the phases of the gait cycle of the two lower limbs, evaluating whether both feet move in a similar and coordinated manner. In subjects with normal feet, gait is almost perfect, with a balanced distribution of loads and use of the body throughout the phases of gait. To our knowledge, there are no studies that analyze the dynamic symmetry index in this pathology; therefore, the aim of this study was to examine the dynamic symmetry index in the inferior limbs by comparing individuals with and without HL using a plantar pressure platform. This study was conducted using the same protocol of Becerro de Bengoa Vallejo et al. [28] for recording time, percentages, and frames from the subjects.

According to the results of our study, we can observe significant differences in the percentage of load supported by both feet between healthy subjects and subjects with hallux limitus. In cases with HL, it was observed that the load is greater in the forefoot and laterally, with even the lateral symmetry index being significant, while subjects with normal feet bear more load at the medial and posterior levels. Also, the effect size for medial load in the lower left limb (%) for the control group and lateral load in the lower left limb (%) for the case group are very large. These results match the *p*-values shown in the output of the ANOVA (Table 2). Therefore, the presence of HL resulted in a decrease in the symmetry of the gait, since the subjects compensated for the lack of hallux function, which led to an uncoordinated gait pattern, evidenced by the variability in the distribution of the load between the feet of both groups during walking. We also found significant differences in the symmetry index in the initial contact phase and in the flatfoot phase, but not in the forefoot phase, which could be justified by this lack of mobility in dorsal flexion of the hallux, which makes propulsion difficult, causing an inefficient transfer of body weight and increasing it on the lateral part of the foot. These results are consistent with previous studies that have analyzed the influence of HL on gait. Zammit et al., in their study that analyzes how osteoarthritis and hallux limitus range of motion alter plantar pressure distribution and affect gait, conclude that subjects with hallux limitus presented greater pressure in the forefoot due to a lack of hallux propulsion, causing other structures to bear more load without being prepared for it [34]. On the other hand, Van Gheluwe et al., in their study that evaluated the effects of HL on plantar pressure and foot kinematics, also conclude that the presence of HL resulted in a decrease in gait symmetry, as subjects compensated for the lack of hallux function, leading to an uncoordinated gait pattern between both feet, affecting the temporal asymmetry of the phases of the gait cycle [16]. These studies therefore corroborate our results, which show that limited hallux flexion affects body forward motion and stability, altering propulsion and walking efficiency. In addition, it contributes to the need for compensation in other structures of the foot and body, which can increase the risk of developing problems in other areas such as the knees, hip, and back.

In our study, subjects with HL were overweight (77.49 ± 15.58 kg) and had a high BMI (28.98 ± 6.27 kg/m^2^), a condition that can affect gait, leading to instability and causing the appearance or worsening of foot problems. Hershkovich et al. performed a study about epidemiology and risk factors for chronic ankle instability. The principal findings reported that instability severity was associated with subjects with increased BMI and greater body height [35]. On the other hand, Kim et al., in their study investigating variations in dynamic balance control across walking speeds in obese persons, found that these variations cause gait instability, suggesting that maintaining mediolateral stability during walking is more difficult in obese adults and highlighting the biomechanical relationship that exists between obesity and gait instability [36]. Taking into account the observations made by these authors, this may have influenced the results of our study, since based on our findings, there were statistically significant variations in the symmetry index lateral load between the control group and the case group (*p* = 0.023), with subjects with HL exhibiting greater instability in foot lateral load; in addition, the medial and lateral load in the lower right and left limb also showed a statistically significant result (right *p* = 0.016) (left *p* = 0.018), which may be conditioned by the overweight that these subjects presented. These results, in addition to highlighting the growing public health problem posed by obesity, establish that we must find a way to reduce the risk of falls and increase physical activity in these subjects.

Another study that supports the alterations in the lateral load symmetry index found in our research is the one carried out by Ko et al., who demonstrated statistically significant differences in stride width between normal and overweight subjects [37]. Cau et al., in their study, observed that the change in posture in individuals with weight gain from the stance phase to the initial contact phase generates alterations in mediolateral movements, increasing the center of pressure speed, while in front–back movements, it decreases compared to the control group [38]. Based on these findings, we can determine that, in subjects with increased weight, the stride width would be greater and the load would be increased in the lateral area of the foot.

According to research reported by Liu et al., overweight older women, compared to non-overweight women, had more plantar pressure in metatarsal 1 to 5 and in the medial and lateral area of the heel, especially the midfoot and rearfoot [39]. These conclusions are in agreement with our study, which has shown statistically significant results for the symmetry index for the initial and flatfoot contact phases, but we did not find differences in the forefoot contact phase. This can also be explained based on the study by De Castro et al. [24], in which they observed that overweight patients presented less load in the forefoot area during walking than the control group.

These altered patterns could contribute to joint pain, muscle fatigue, and a higher incidence of falls and injuries. The identification of these gait changes in obese adults highlights the importance of implementing rehabilitation programs and mobility exercises to improve gait symmetry and efficiency [40]. Gait asymmetries lead to functional limitations in several aspects of life, including walking, climbing stairs, and maintaining balance [41]. Furthermore, gait asymmetry in individuals with conditions like osteoarthritis or musculoskeletal injuries can limit their ability to perform daily tasks like shopping, cleaning, or even standing for prolonged periods. This functional decline is not only due to the inefficiency in movement but also due to pain or discomfort associated with compensatory patterns. The long-term implications of these compensatory adaptations can also lead to progressive joint damage. Considering the above, research on gait asymmetry seems to be important, because cyclic uneven movement repeated daily for hours can cause the appearance of low back pain or problems in the knee and hip joints. In this way, if gait asymmetry is detected early, adequate rehabilitation and effective treatment can be designed for a patient to recover their gait pattern.

Our study has limitations; it could be interesting to take into account other factors that are considered to increase the risk of presenting this pathology when selecting participants with HL, such as age or sex, which in this study has proven to be relevant and therefore should also be taken into account in future works, since in this research, due to the small size of the sample, we have not been able to classify the subjects by their BMI. A more balanced sample by sex could also be obtained to analyze the possible differences. Including a diverse sample of patients from different countries would strengthen the research by improving generalizability and identifying cultural variations in the observed association. Additionally, it could help uncover underlying mechanisms, especially since knee osteoarthritis and limb length discrepancy were not considered as potential confounders in this study. On the other hand, for future research, it would also be interesting to add one more group of cases, for example, a group with unilateral pathology, and thus also be able to compare the differences between the group with unilateral or bilateral pathology compared to the group with normal feet. Therefore, going deeper into these aspects would be interesting since the etiology of HL remains unclear, and this could help to determine the relationship that may exist between the mechanisms that cause its appearance. Based on this, in future works we will continue to increase the sample size and the number of groups and, in this way, be able to include more variables that can further enrich our research and continue to provide information on those aspects that can influence instability and gait alteration in order to be able to act preventively, modifying habits or compensating for the alteration early, and thus be able to improve the quality of life of these subjects.

## 5. Conclusions

The subjects with weight gain and bilateral HL presented changes in the symmetry index, specifically in the lateral load and in the initial and flatfoot contact phases, which lead to greater instability in these subjects compared to the control group. We believe that early identification of these changes can help improve the quality of life of these subjects and avoid future complications.

## Figures and Tables

**Table 1:** Main characteristics of total sample with and without bilateral hallux limitus.

Characteristics	Total Sample (*n* = 70) Mean ± SD (Range)	Case Group (*n* = 35) Mean ± SD (Range)	Control Group (*n* = 35) Mean ± SD (Range)	*p*-Value
Age (years)	25.80 ± 4.46 (21–39)	26.09 ± 4.27 (21–36)	25.51 ± 4.69 (21–39)	0.138 †
Weight (kg)	72.26 ± 14.28 (53–98)	77.49 ± 15.58 (56–98)	67.09 ± 10.73 (53-89)	**<0.007 †**
Height (cm)	164.96 ± 8.41 (150–185)	163.80 ± 7.98 (152–185)	166.11 ± 8.79 (150–185)	0.205 †
BMI (kg/m^2^)	26.62 ± 5.48 (19.00–39.26)	28.98 ± 6.27 (21.08–39.26)	24.27 ± 3.20 (19.00–30.44)	**<0.002 †**
Sex, male/female (%)	11/59 (15.7/84.3)	7/28 (20/80)	4/31 (11.4/88.6)	0.256 ‡
Foot size (N)	38.97 ± 2.41 (36–46)	38.78 ± 2.11 (36–44)	39.17 ± 2.69 (36–46)	0.523 †

Abbreviations: kg, kilogram; cm, centimeter; m^2^, square meter; %, percentage; SD, standard deviation; N, number. † Mann–Whitney U test was used. ‡ Fisher’s exact test was used. In all the analyses, *p* < 0.05 (with a 95% confidence interval) was considered statistically significant (**bold**).

**Table 2:** Main outcome measurements of symmetry index analysis of total sample with and without bilateral hallux limitus.

Characteristics	Total Sample (*n* = 70) Mean ± SD (Range)	Case Group (*n* = 35) Mean ± SD (Range)	Control Group (*n* = 35) Mean ± SD (Range)	*p*-Value	Partial Eta Squared
Anterior load in the LLL (%)	48.58 ± 3.79 (36.80–53.70)	49.47 ± 3.52 (42.80–53.70)	47.68 ± 3.90 (36.80–53.20)	0.076 †	0.132
Posterior load in the LLL (%)	51.42 ± 3.79 (46.30–63.20)	50.53 ± 3.52 (46.30–57.20)	52.32 ± 3.90 (46.80–63.20)	0.076 †	0.132
Medial load in the LLL (%)	44.55 ± 4.20 (36.70–52.80)	43.11 ± 4.06 (36.70–49.40)	45.98 ± 3.88 (40.00–52.80)	**0.018 †**	0.229 *
Lateral load in the LLL (%)	55.47 ± 4.20 (47.20–63.30)	56.89 ± 4.06 (50.60–63.30)	54.05 ± 3.90 (47.20–60.00)	**0.018 †**	0.228 *
Left foot FFP (ms)	268.24 ± 63.02 (162.70–442.00)	274.25 ± 60.91 (176.20–367.00)	262.23 ± 65.39 (162.70–442.00)	0.184 †	0.031
Left foot FFCP (ms)	389.58 ± 88.83 (257.30–605.60)	395.59 ± 108.85 (265.30–605.60)	383.57 ± 63.96 (257.30–465.00)	0.769 †	0.013
Left foot ICP (ms)	94.00 ± 31.56 (43.70–166.50)	91.41 ± 32.30 (43.70–166.50)	96.60 ± 31.05 (44.60–154.30)	0.353 †	0.018
Anterior load in the LRL (%)	47.80 ± 4.49 (40.30–54.90)	48.92 ± 4.95 (41.20–54.90)	46.68 ± 3.70 (40.30–52.00)	**0.048 †**	0.109
Posterior load in the LRL (%)	52.20 ± 4.49 (45.10–59.70)	51.08 ± 4.95 (45.10–58.80)	53.32 ± 3.70 (47.80–59.70)	**0.048 †**	0.083
Medial load in the LRL (%)	52.45 ± 5.15 (40.60–61.80)	50.56 ± 5.51 (40.60–56.50)	54.33 ± 4.02 (48.00–61.80)	**0.016 †**	0.015
Lateral load in the LRL (%)	47.55 ± 5.15 (38.20–59.40)	49.44 ± 5.51 (43.50–59.40)	45.67 ± 4.02 (38.20–52.00)	**0.016 †**	0.063
Right foot FFP (ms)	249.46 ± 59.31 (165.80–405.50)	241.13 ± 51.64 (187.20–323.00)	257.78 ± 65.80 (165.80–405.50)	0.359 †	0.079
Right foot FFCP (ms)	390.02 ± 103.00 (34.15–544.00)	417.47 ± 89.10 (268.70–544.00)	362.57 ± 109.73 (34.15–513.20)	**0.011 †**	0.054
Right foot ICP (ms)	95.90 ± 28.89 (42.80–141.80)	98.20 ± 24.61 (54.40–141.40)	93.61 ± 32.83 (42.80–141.80)	0.897 †	0.132
Symmetry index anterior load (%)	94.51 ± 4.63 (80.70–99.80)	95.44 ± 3.48 (89.10–99.80)	93.58 ± 5.44 (80.70–99.10)	0.165 †	0.109
Symmetry index posterior load (%)	95.03 ± 4.10 (82.00–99.80)	95.91 ± 2.66 (91.40–98.80)	94.16 ± 5.05 (82.00–99.00)	0.502 †	0.093
Symmetry index medial load (%)	85.13 ± 5.89 (75.40–99.40)	85.60 ± 5.75 (76.50–99.40)	84.66 ± 6.07 (75.40–99.40)	0.204 †	0.015
Symmetry index lateral load (%)	85.71 ± 6.31 (72.10–99.50)	86.85 ± 6.13 (76.70–99.50)	84.56 ± 6.38 (72.10–99.50)	**0.023 †**	0.063
Symmetry index FFP (%)	86.27 ± 9.62 (64.00–99.90)	82.72 ± 8.59 (64.00–91.50)	89.83 ± 9.36 (73.50–99.90)	**<0.001 †**	0.079
Symmetry index FFCP (%)	90.66 ± 7.47 (69.10–99.30)	89.46 ± 8.45 (69.10–98.50)	91.86 ± 6.24 (79.60–99.30)	0.200 †	0.054
Symmetry index ICP (%)	84.17 ± 14.46 (51.90–100.00)	79.82 ± 14.95 (51.90–96.50)	88.52 ± 12.73 (53.00–100.00)	**0.003 †**	0.132

Abbreviations: ICP, initial contact phase; FFCP, forefoot contact phase; FFP, flatfoot phase; LLL, lower left limb; LRL, lower right limb; Ms, meters per second; %, percentage; SD, standard deviation; † Mann–Whitney U test and ANOVA model used. * Large effect size ≥ 0.14. In all the analyses, *p* < 0.05 (with a 95% confidence interval) was considered statistically significant (**bold**).

## Data Availability

The dataset supporting the conclusions of this article is available upon request to daniellopez@udc.es in the Research, Health and Podiatry Group, Department of Health Sciences, Faculty of Nursing and Podiatry, Industrial Campus of Ferrol, Universidade da Coruña, 15403, Ferrol, Spain.
